# Solitary fibrous tumor of the pineal gland: a case report and review of the literature

**DOI:** 10.3389/fonc.2024.1392540

**Published:** 2024-08-08

**Authors:** Yixiao He, Pengchen He, Anqun Wang, Yuzhu Ji, Gang Xie, Lili Zou

**Affiliations:** ^1^ Department of Pathology, Mianyang Central Hospital, School of Medicine, University of Electronic Science and Technology of China, Mianyang, Sichuan, China; ^2^ Department of Neurosurgery, Mianyang Central Hospital, School of Medicine, University of Electronic Science and Technology of China, Mianyang, Sichuan, China

**Keywords:** solitary fibrous tumor (SFT), pineal gland, central nervous system, mesenchymal tumor, immunohistochemistry

## Abstract

Solitary fibrous tumor (SFT) is a type of fibroblastic neoplasm that can occur in various parts of the body, with SFT of the pineal gland being exceedingly rare. We report the case of a 58-year-old male presenting with recurrent hiccups, acid reflux, and headache. Magnetic resonance imaging revealed an occupying lesion in the pineal region, suggestive of a neoplastic process. Intraoperatively, the lesion was located in the pineal region, exhibiting a grayish-red color, and was largely resected. Pathological examination confirmed the diagnosis of solitary fibrous tumor (CNS WHO Grade 1). Postoperatively, the patient was supplemented with radiotherapy, and long-term follow-up showed no signs of recurrence or metastasis.

## 1 Introduction

Solitary fibrous tumor (SFT) is a fibroblastic mesenchymal neoplasm (1) that can arise in any part of the body, with a predilection for the pleura. A subset of cases may occur outside the pleural location, such as in peripheral soft tissues, the retroperitoneal space, the mediastinum, and the head and neck regions (2). Primary central nervous system (CNS) SFTs are exceedingly rare, accounting for less than 1% of all CNS tumors, with those occurring in the pineal gland being exceptionally rare. Herein, we present a case report detailing the clinical, radiographic, and pathological characteristics of a pineal SFT and provide a review of the relevant literature.

## 2 Case report

### 2.1 Clinical presentation

A 58-year-old male patient presented with recurrent hiccups and acid reflux for half a year, and headache for half a month, each episode lasting for several days, without vomiting or dizziness. Neurological examination revealed no positive signs, and there was no history of related neurological or other specific diseases in the patient or his family.

### 2.2 Imaging and laboratory examinations

**Figure 1:**
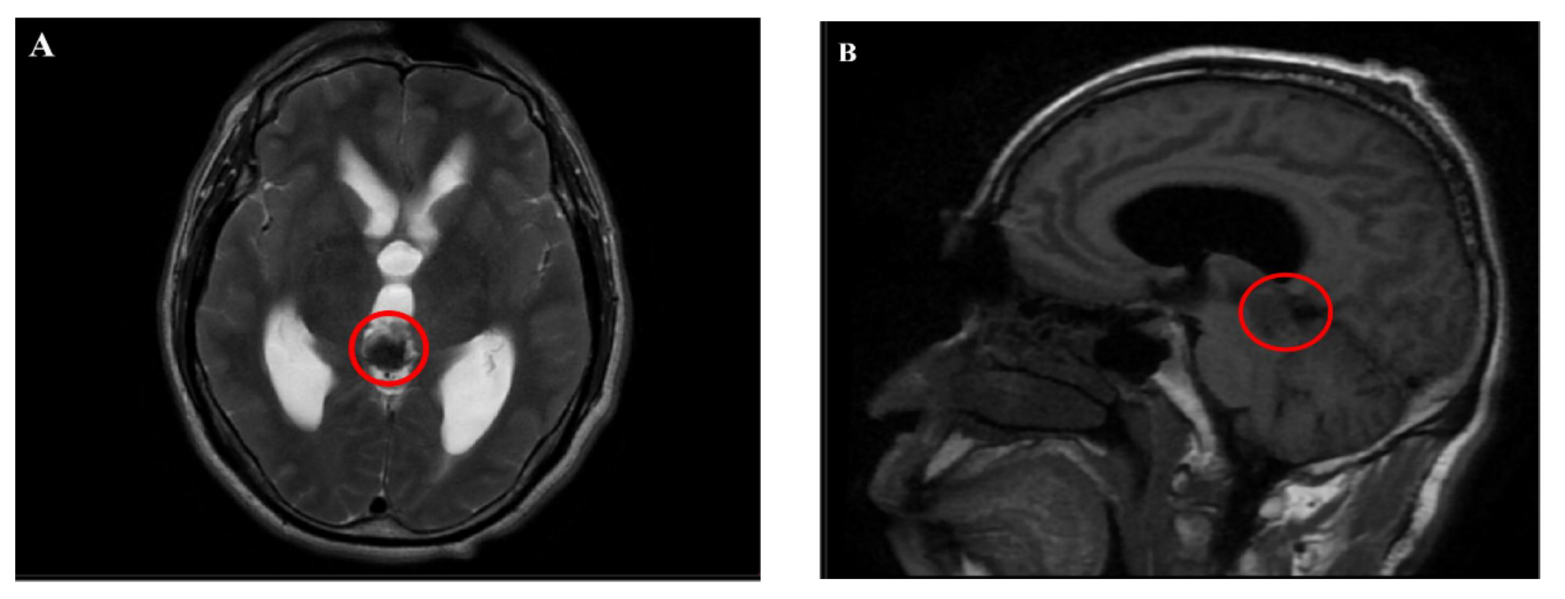
**(A, B)** MRI scanning revealed an abnormal signal nodular shadow in the pineal region.

**Figure 2:**
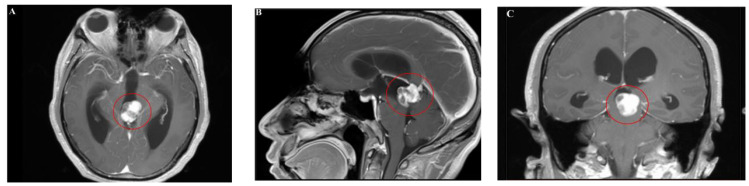
**(A–C)** Enhanced MRI showed marked heterogeneous enhancement.

Magnetic resonance imaging (MRI) showed an abnormal signal nodule in the pineal region, measuring approximately 2.6×2.4×1.9 cm, with markedly heterogeneous enhancement on contrast scans. Compression of the third ventricle and the cerebral aqueduct was observed, leading to obstructive hydrocephalus in the supratentorial ventricles, suggestive of a neoplastic lesion (**Figures 1**, **2**). Chest and abdominal computed tomography (CT) scans were unremarkable, and preoperative serological tests were within normal limits.

### 2.3 Intraoperative findings

On June 13, 2023, the patient underwent resection of the pineal region lesion via a suboccipital-supracerebellar approach. No abnormalities were observed in the scalp and skull of the operative area, and intracranial pressure was not elevated. The lesion was located in the pineal region, with a grayish-red color, and the tumor was estimated to be approximately 3 cm x 2 cm x 2 cm in size, hard and tough with ill-defined borders, and richly vascularized. It was closely adherent to the superior cerebellar hemisphere, the posterior part of the third ventricle, and involved the terminal end of the great cerebral vein. The tumor compressed and pushed the quadrigeminal plate area of the brainstem and the superior part of the cerebellum inward and downward. Most of the tumor was resected, leaving only a portion approximately 0.5 cm tightly adhering to the terminal end of the great cerebral vein. The surgical procedure was uneventful, intraoperative bleeding is approximately 400ml.

### 2.4 Pathological examination

**Figure 3:**
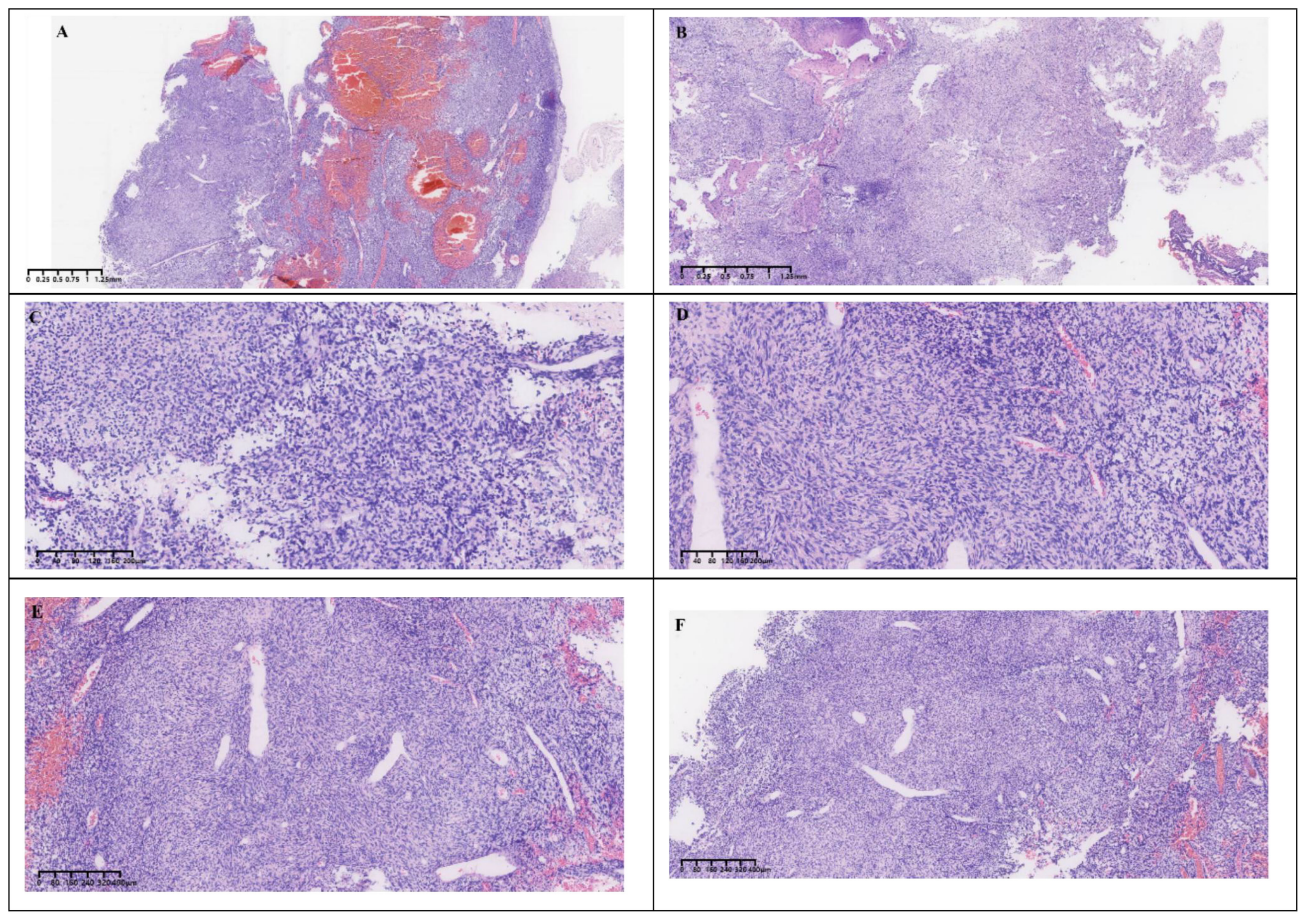
Histological morphology. **(A, B)** The cells were rich and grew in a solid, sheet-like manner with alternating densities. **(C, D)** The tumor cells were short spindle-shaped and oval, without a specific arrangement pattern. **(E, F)** The background showed staghorn vessels and collagen.

**Figure 4:**
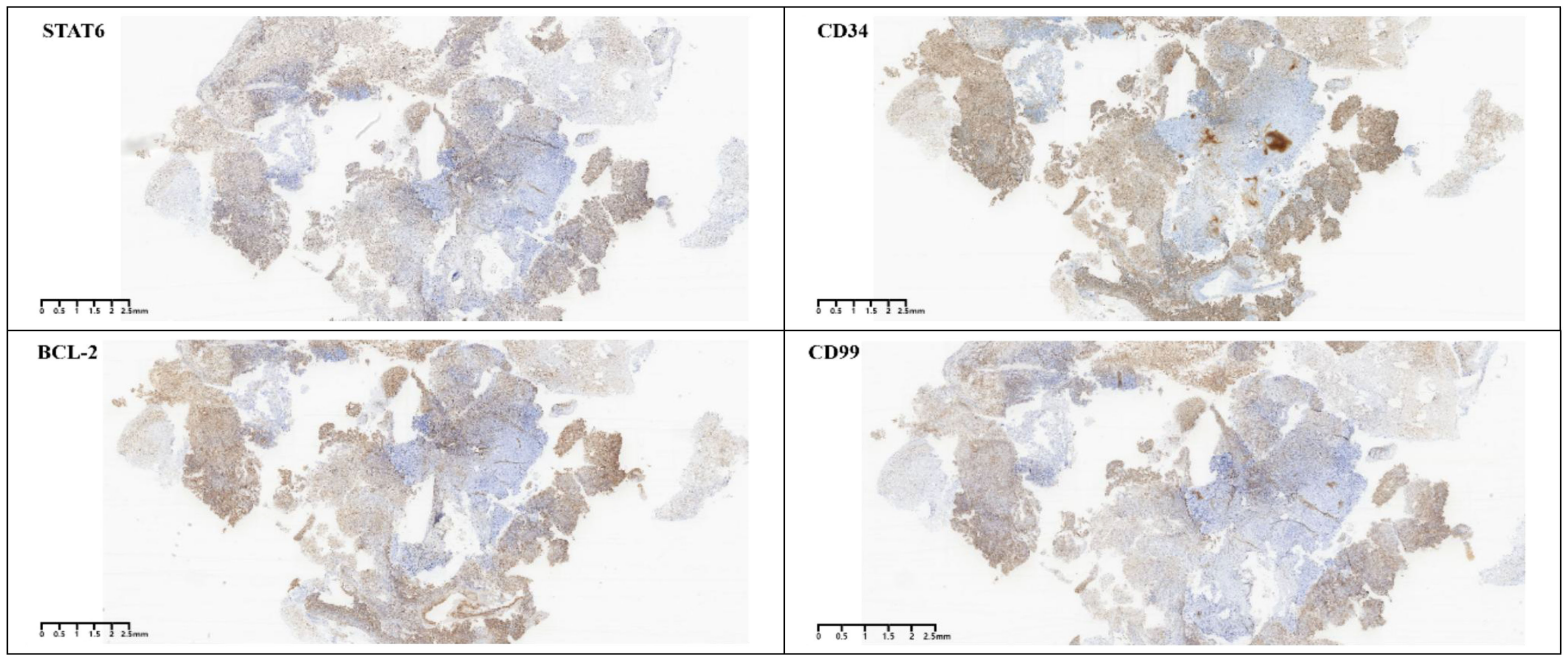
Immunohistochemistry. Tumor cells positive for STAT6, CD34, Bcl-2, CD99.

Gross postoperative examination revealed a gray-white fragmented tissue measuring 3 cm × 2.5 cm × 1 cm. Microscopically, the tumor was cellular with a solid sheet-like growth pattern, alternating between dense and sparse areas. The neoplastic cells were short spindle-shaped or oval, without a specific arrangement, exhibiting a bland morphology with fine chromatin, and no necrosis was observed. The stroma contained staghorn blood vessels and collagen (**Figure 3**). Immunohistochemistry demonstrated the tumor cells to be positive for CD34, CD99, STAT6, and BCL-2 (**Figure 4**), while negative for S-100, CD45, P-CK, CD56 (focally positive), Sy, GFAP, SALL4, OCT3/4, and Desmin. The Ki-67 proliferation index was approximately 2%. According to literature reports, gross examination of SFTs typically reveals irregular or nodular masses with a solid cut surface, gray-white to gray-brown, of medium to soft consistency, sometimes with myxoid changes, and malignant cases often exhibit hemorrhage, necrosis, and cystic degeneration. Based on the 2016 WHO classification of tumors of the central nervous system, the typical pathological features of SFT include spindle-shaped or oval tumor cells that are densely packed with a bland morphology and fine chromatin, arranged in alternating dense and sparse patterns, with a background of staghorn blood vessels interspersed with collagen-rich tissue. Additionally, rare cases may present with histological patterns such as papillary structures, melanocytic differentiation (3), and lipomatous metaplasia. Malignant cases often show increased cellular density, marked atypia, increased mitotic figures, or necrosis. SFTs are graded from grade 1 to grade 3 (CNS WHO grades 1 to 3), with grade 1 characterized by sparse tumor cells, absence of mitotic figures and necrosis, and a stroma often containing hyalinized collagen fibers; grade 2 by increased tumor cell density, possible atypia, and visible mitotic figures (<5 per 10 HPF); and grade 3 by abundant tumor cells, high density, marked atypia, and visible mitotic figures (>5 per 10 HPF), with necrosis (4). According to these criteria, the final pathological diagnosis of this case was a CNS WHO grade 1 solitary fibrous tumor of the pineal region.

### 2.5 Follow-up

**Figure 5:**
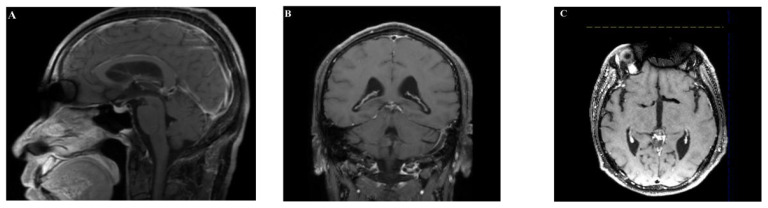
**(A–C)** Postoperative follow-up magnetic resonance imaging.

The patient had an uneventful postoperative recovery without complications and was discharged on July 1, 2023. Subsequently, the patient received radiotherapy at our institution. Long-term follow-up(4 months after surgery) with imaging studies showed no evidence of tumor recurrence or metastasis (**Figure 5**).

## 3 Discussion

### 3.1 Central nervous system SFT origin

Solitary fibrous tumor (SFT) was first reported by Klemperer and Robin in 1931 (5), and initially classified separately from hemangiopericytoma (HPC). In 2021, the World Health Organization (WHO) removed the term “HPC” and collectively referred to the entity as “SFT”. SFTs can occur throughout the body and affect individuals of any age, commonly presenting in adults aged 20 to 70 years, with no significant gender predilection. SFTs of the central nervous system (CNS) are relatively rare, with the earliest description as a distinct entity tumor by Carneiro et al. in 1996. Regarding the origin of CNS SFTs, early studies by Cummings et al. (6) suggested that they may arise from CD34-expressing fibroblastic cells of the outer dural border layer, which are attached to the arachnoid membrane beneath the dura mater. Given that the choroid plexus stroma contains arachnoid matrix components and soft meninges, it is hypothesized that this may be the reason for the occurrence of intraventricular SFTs. However, Kim et al. (7)proposed that CNS SFTs may originate from the mesenchymal components of the cerebral vasculature, thus further investigation is needed to elucidate the origin of CNS SFTs. Historically, most CNS SFTs were believed to occur intracranially, most commonly located along the falx cerebri, the occipital lobe, and the dura mater of the spinal cord, cerebellar tentorium, and cerebellopontine angle, often adhering to the meninges. However, SFTs in the pineal region are extremely rare. In this article, a PRISMA analysis process was conducted, identifying nine relevant literature reports (8–16) (**Table 1**; **Supplementary Figure 1**), including one video abstract (16), and another study reported a biphasic tumor composed of pilocytic astrocytoma and a small amount of pineal region anaplastic solitary fibrous tumor (15).

**Table 1:** Summary of reports of solitary fibrous tumor of the pineal gland.

Author	Year	Age, Sex	Clinical Symptoms	Neurological examination	Size	treatment	IHC(positive)	WHO grading	Follow-up
J Zhang (13)	2010	49/F	headache, slowly progressive weakness of the right lower extremities, upgaze palsy	a positive right-sided Babinski sign.	5.4×5.1×4.1 cm	Surgery	CD34,CD99, vimentin	Grade 3	living with the disease at 10 months post-surgical follow-up
Wen G (10)	2014	44/F	Headache, dizziness	Negative	2.2×2.5×2.1 cm	Surgery	Bcl-2,CD34, CD99,Vimentin	Grade 1	no evidence of recurrence six months after surgery.
		52/M	numbness and weakness in all four extremities	hypalgesia, hypothermesthesia, hypopselaphesia of the whole body	2.3×1.8×2.7 cm	Surgery	CD34, Vimentin	Grade 1	lost to follow-up from three months after operation.
Huang HJ (11)	2017	22/M, 45/F	Headache,nausea and hearing loss	–	–	Surgery and radiotherapy	CD34,CD99, STAT6,Bcl-2, Vimentin	Grade 3	The male patient experienced two recurrences post-surgery. The female has shown no recurrence and metastasis.
Michele M (12)	2018	72/M	Severe headache, seizure and gait instability, poor concentration and attention.	–	3.7×3.3×3.6 cm	Surgery	Bcl-2, CD34, CD99, Vimentin	–	–
Y Wang (8)	2019	35/F	progressive headache with double vision, memory impairment and unsteady gait	walked slowly despite intact muscle strength, and had difficult with up-gazing.	5.2×3.5×3.2cm	Surgery and radiotherapy	vimentin, CD99,Bcl-2	Grade 3	The patient experienced a recurrence followed by intracranial metastasis, and was followed up routinely for 7 months with no signs of recurrence.
Oketani H (9)	2022	80/M	lightheadedness, headache, vomiting	Negative	3.8×3.1×3.8cm	Surgery and radiotherapy	CD34,CD99, STAT6	Grade 1	No recurrence and metastasis five months after the operation,
Demir MK (14)	2023	25/M	–	–	–	Surgery and radiotherapy	–	Grade 3	The patient experienced a recurrence followed by widespread intracranial intra-axial and extra-axial metastases.

### 3.2 Clinical manifestations

The peak incidence of CNS SFTs occurs between the ages of 40 and 50 years, with a higher prevalence in males compared to females. The vast majority of these tumors arise in the meninges of the adult supratentorial brain or spinal cord, with varying degrees of invasion into the CNS parenchyma, nerve roots, and skull base, with some occurring in the spinal cord. Clinical presentations vary depending on the location and size of the tumor. Obstruction of cerebrospinal fluid (CSF) circulation by the tumor can lead to increased intracranial pressure, manifesting as symptoms of intracranial tumors such as dizziness, headache, nausea, and vomiting. Some patients may also present with extensive intracranial hemorrhage and paraneoplastic syndromes associated with hypoglycemia. In this case, the patient is a 58-year-old male with symptoms of headache, hiccups, and acid reflux. MRI revealed a tumor located in the pineal region.

### 3.3 Immunohistochemistry

SFTs of the CNS often adhere to the dura mater, presenting as round or oval-shaped, well-demarcated masses, similar to meningiomas. This similarity makes it challenging to differentiate SFTs from meningiomas on imaging studies. Therefore, the combination of histological morphology and immunohistochemistry is particularly important for the diagnosis of SFTs. Previous studies on SFTs have shown that they exhibit a characteristic immunohistochemical phenotype, with diffuse positivity for CD34, vimentin, CD99, and Bcl-2, while epithelial membrane antigen (EMA) and S-100 are usually negative. The most distinctive feature compared to other tumors is the high expression of STAT6 protein, which is highly sensitive and specific (17). ALDH1 and GRIA2 are specific antibodies for SFTs that have been proposed in recent years in addition to STAT6. Studies have shown that the combination of nuclear positivity for STAT6 and cytoplasmic positivity for ALDH1 is the most sensitive and specific set of markers for the differential diagnosis of SFTs (18). Therefore, the classic histological features combined with the typical immunohistochemical phenotype are helpful for accurate diagnosis. In this case, the tumor cells were short spindle-shaped and oval, with a bland morphology and irregular arrangement. The background showed staghorn blood vessels and collagen, without necrosis. The immunohistochemical results showed positivity for CD34, CD99, STAT6, and BCL2, with a low Ki-67 proliferation index, consistent with the diagnosis of a CNS WHO grade 1.

### 3.4 Differential diagnosis

SFTs of the CNS share many morphological commonalities or similarities with other spindle cell tumors that arise in the dura mater, necessitating differential diagnosis. The main differential diagnoses include (1): Meningiomas, especially the fibrous type, which are clinically and radiologically very similar to SFTs. However, meningiomas typically express epithelial membrane antigen (EMA) positively, while CD34 and STAT6 are negative. (2) Schwannomas: Often located in the cerebellopontine angle and spinal canal, histologically characterized by areas of sparsity and density, with palisading arrangement, and typically diffusely express S-100 protein and SOX10, without expression of STAT6 or CD34. (3) Monophasic synovial sarcoma: Like SFTs, it exhibits features of hemangiopericytoma, but monophasic synovial sarcoma expresses EMA and TLE1, does not express STAT6 or CD34, and has a specific SS18-SSX1/2/4 fusion gene. (4) Dural-based Ewing sarcoma/peripheral primitive neuroectodermal tumour shares the hypercellularity and CD99 positivity of haemangiopericytoma, but lacks nuclear STAT6 staining and is characterized by EWSR1 gene rearrangement in the great majority of cases.

### 3.5 Advances in molecular pathology research

In the field of molecular genetic testing, studies have identified that the most characteristic feature of the majority of SFTs is the presence of the NAB2-STAT6 gene fusion, which has high sensitivity and specificity for the diagnosis of SFTs. Both the NAB2 and STAT6 genes are located on 12q13. Some researchers, through whole-genome sequencing studies, have found that most SFTs exhibit translocations of the NAB2 and STAT6 genes, resulting in the formation of the NAB2-STAT6 fusion gene (19–21). Robinson et al. demonstrated through integrative sequencing studies that almost all SFTs (including benign and malignant) have fusion mutations of NAB2 and STAT6 in the tumor pathway (21). The underlying mechanism is that in the context of SFTs, the NAB2-STAT6 fusion gene confers an activation domain from the signal molecule STAT6 to the NAB2 fusion, thereby activating the STAT6 domain. This activation domain transforms the transcriptional repressor (NAB2) into a potent transcriptional activator of EGR1 (NAB2-STAT6), leading to the transition from an EGR repressive state to an activated state in the structural domain of NAB2. This induces the expression of EGR1 target genes, which have a biological effect, ultimately forming a feed-forward loop that drives tumor progression. Therefore, the detection of the NAB2 and STAT6 fusion gene can facilitate the auxiliary diagnosis of SFTs. In recent years, research has discovered several different fusion sites for NAB2 and STAT6, and SFTs with different fusion types exhibit distinct clinical and pathological characteristics. Tai et al. conducted an analysis of the NAB2-STAT6 gene fusion status in 73 cases of SFTs and found that the most common fusion type was the fusion of exon 4 of the NAB2 gene with exon 2 of the STAT6 gene (NAB2ex4-STAT6ex2), followed by NAB2ex6-STAT6ex16 and NAB2ex6-STAT6ex17. Compared to the latter two fusion types, the NAB2ex4-STAT6ex2 fusion subtype was significantly associated with several clinical and pathological characteristics of SFTs: including intrathoracic occurrence, older age of onset, extensive fibrous sclerotic stroma, and a lower number of mitotic figures, but the relationship with overall disease survival remains unclear (22). In this study, out of 19 meningeal SFTs, only 2 cases exhibited the NAB2ex4-STAT6ex2 fusion, suggesting that the NAB2-STAT6 fusion types in meningeal SFTs differ from those at other sites. Nakada et al. conducted a retrospective review of 546 SFTs that underwent NAB2-STAT6 fusion gene analysis and explored the differences among various gene variants. The most frequent of these were NAB2 exon 6-STAT6 exon 16/17/18 and NAB2 exon 4-STAT6 exon 2/3, with the former occurring most frequently in SFTs in meninges, soft tissues, and head and neck. There were no significant differences observed between the histological features of SFTs and the types of fusion gene variants. The follow-up analysis of SFTs revealed in meninges and soft tissue, SFTs with the NAB2 exon 6-STAT6 exon 16/17/18 tended to recur more frequently than SFTs with the NAB2 exon 4-STAT6 exon 2/3 (23). Consistent with the study by Nakada et al., Fritchie et al. examined the NAB2-STAT6 fusion types in 99 cases of meningeal SFTs and found that the most common was NAB2ex5/6/7-STAT6ex16/17, followed by NAB2ex4-STAT6ex2/3. In this study, it was observed that the NAB2-STAT6 fusion types were not associated with prognosis but were related to phenotype (24). In addition to the NAB2-STAT6 gene fusion, other studies have discovered that SFTs highly express the ALDH1 (25) and GRIA2 (26) genes.

In summary, the types of NAB2-STAT6 gene fusions in CNS SFTs exhibit differences compared to SFTs in other locations. Although different fusion types may correlate with distinct clinical and pathological characteristics, the relationship between STAT6 and overall survival remains unclear. Therefore, to elucidate the association between STAT6 and prognosis in CNS SFTs, it is necessary to collect clinical and pathological data, including annual follow-up information.

### 3.6 Treatment and prognosis

Most SFTs of CNS are benign in nature, although a minority are malignant and carry the risk of local recurrence and metastasis. A survey study of 220 CNS SFTs by Michele Bisceglia et al. indicated that approximately 5.8% of CNS SFTs are malignant. Additionally, a small subset of intracranial SFTs undergo malignant transformation and delayed extracranial metastasis, commonly to the lungs, liver, and other sites. Therefore, early and accurate diagnosis and treatment are particularly crucial. The primary first-line treatment is surgical resection, in combination with radiotherapy, which can significantly prolong the time to recurrence and overall survival. In this case, the patient underwent radiotherapy postoperatively, and long-term follow-up has not revealed any recurrence or metastasis to date.

## Data availability statement

The datasets presented in this article are not readily available because of ethical and privacy restrictions. Requests to access the datasets should be directed to the corresponding author/s.

## Ethics statement

The studies involving humans were approved by the Ethics Review Committee of Mianyang Central Hospital. The studies were conducted in accordance with the local legislation and institutional requirements. The participants provided their written informed consent to participate in this study. Written informed consent was obtained from the individual(s) for the publication of any potentially identifiable images or data included in this article.

## Author contributions

YH: Writing – review & editing, Writing – original draft, Resources. PH: Writing – review & editing, Resources, Data curation. AW: Writing – review & editing, Resources, Data curation. YJ: Writing – review & editing, Validation, Supervision. GX: Writing – review & editing, Validation, Supervision. LZ: Writing – review & editing, Validation, Data curation.
