# Supplementary material for: Solitary fibrous tumor of the pineal gland: a case report and review of the literature
Source: Front Oncol. 2024 Aug 8;14:1392540. doi: 10.3389/fonc.2024.1392540 (PMC11338914; doi:10.3389/fonc.2024.1392540)
Supplement: Supplementary file 1 [file DataSheet_1.docx]

**Identification of studies via databases**

Records removed *before screening*:

Duplicate records removed (n = 3)

Records marked as ineligible by automation tools (n = 0)

Records removed for other reasons (n = 0)

**Identification**

Records identified from:

Pubmed (n = 15)

WANFANG（n=1）

**Included**

**Screening**

Reports not retrieved

(n =0 )

Records assessed for eligibility

(n =13 )

Reports excluded:

Reason 1 (n = 1)

Reason 2 (n = 1)

Reason 3 (n = 1)

Reason 4 (n = 1)

Studies included in review

(n = 9)

Reports of included studies

(n = 9)

Records sought for retrieval

(n = 13)

Records excluded

(n = 0)

Records screened

(n =13)

Figure 1 the study selection process.

The analysis of this systematic review followed the Preferred Reporting Items for Systematic Reviews and Meta-Analysis (PRISMA) statement guidelines.

1. Literature Search Strategy

We conducted a systematic literature review to identify all publications related to solitary fibrous tumors (SFTs) of the pineal gland. Initially, several databases were considered, including VIP, Wanfang, and PubMed. However, due to issues of search result redundancy, available filtering options, and the required scope of coverage, the search was ultimately limited to the Wanfang and PubMed databases. The search was performed using the "all fields" search option on both platforms. The search terms included combinations of[ "solitary fibrous tumors" AND "pineal gland" ]or ["solitary fibrous tumors" AND "pineal region"].

2. Literature Selection, Exclusion, and Data Extraction

Two researchers conducted an extensive search of the available literature up to June 2024, without applying any filter conditions to the search results. Due to the limited number of publications retrieved, the researchers reviewed the full text of each article for screening, excluding the following studies: (1) simplified overviews of the classification of central nervous system tumors, (2) overviews of common tumors in the pineal region, (3) classifications of primary central nervous system tumors in adults, and (4) studies where the SFT did not occur in the pineal gland. All four excluded articles were systematic reviews, not case reports, with one being a case report that was not relevant to the topic due to the SFT not being located in the pineal gland.

The researchers were not swayed by the authors, institutions, and titles of the studies not included in the review. Two researchers extracted information from the included articles regarding clinical manifestations, imaging characteristics, pathological diagnosis, treatment, and prognosis.

3 Results

Figure 1 outlines the study selection process. Initially, a total of 16 articles were retrieved, comprising 15 articles in English and 1 in Chinese. After excluding three duplicate articles, the remaining articles were filtered through the exclusion criteria, resulting in the inclusion of 9 studies for the final in-depth review.
